# Longevity of Dentists

**Published:** 1900-06

**Authors:** Waldo E. Boardman

**Affiliations:** Boston, Mass.


### LONGEVITY OF DENTISTS.[1]

BY WALDO E. BOARDMAN, D.M.D., BOSTON, MASS.

In casting about for material in the preparation of a paper, the writer was aware of the irksome task of finding something new and interesting. He has, however, selected the above-named topic, and, without elaboration, will endeavor to give some facts, which are new as far as he has been able to learn.

A great deal of discussion has been given in dental societies relative to the short life of dentists as a body, by reason of the physical and mental high pressure to which they are subjected; that their lives are imperilled to a greater extent than are those engaged in other professional careers; that they are overworked and labor in poorly ventilated quarters, irregular in their habits, inhale the breath of patients, and in the position at the chair are stooped, cramped, and strained, and often for hours maintain such posi-

[1] Read before the Harvard Odontological Society, December 7, 1899.

tions; that they labor too many hours, do not take the necessary recreation, too few if any vacations, etc,—all tending to nerve-strain, so frequently referred to in discussions of the questions pertaining to our calling.

We are subjected to the exacting duties above depicted, many lead sendentary and practically inactive lives, inhale fetid breaths and exhalations from the bodies of patients, some of whom may not be scrupulously neat and clean; others are weak and nervous and come to our chairs in fear and trembling, which adds to the burden put upon our nervous force.

The exacting duties of dentists have often called forth from my clientele remarks upon the unhealthy duties of our professional calling, and they have cited in support of their contention much of which has heretofore been mentioned. It is the general opinion of the laity that we, as professional men, are pursuing an unhealthy occupation.

It is not my purpose here to refute by argument what has been noted thus far, but rather to present facts bearing upon this subject for your consideration, and thought by the speaker to be quite conclusive that dentists, as a body, do live the average length of life allotted to those engaged in other professional pursuits. No doubt we labor under poor hygienic conditions comparable with the dignified callings of law, divinity, and medicine, for we are at all times in contact with varying classes of individuals under different degrees of hygienic ideas. After all, do not dentists in many instances live to a green old age? Many in and about Boston might be cited in support of our contention.

Several years ago the writer was delegated to prepare for publication a catalogue of past students of a professional school which covered a period of three decades, and in so doing he had not only to ascertain facts of those past living students, but of those who had died, and the causes of death. This set him to thinking upon this subject. Still further, he has made search through records of the past fifty years and gathered statistics of all recorded deaths of dentists given by the dental journals, and herewith presents part of the data obtained.

Many believe that consumption is the great destroyer, while others believe Bright's to be the chief disease.

Is the dental profession an unhealthy one? Do dentists live as long as those engaged in other professions, and do the following facts warrant us in believing we are following an unhealthy occupation?

As far as I have been able to verify from the records of the past fifty years, the facts are found to be very different from what was anticipated, for it was supposed that consumption was practically the principle cause of dental mortality.

From the records of between five hundred and six hundred dentists who have died, I have the statistics of sixty-five diseases, eleven of them named below and divided as follows:

Heart disease, 53; consumption, 42; Bright's, 27; pneumonia, 21; typhoid fever, 20; paralysis, 19; accidents, 16; cerebral affections, 13; stomach diseases, 11; cancer, 5; suicides, 5; no disease given, 119. Total, 351.

Their ages ranged from twenty-two to one hundred years. Of one hundred and seventy-six dentists whose years of practice are known, I have figured as six thousand and twenty-nine, or an average of over thirty-four years in practice.

Table of number of deaths, showing average age by decades, and average age of the total:

| Age. | Number of Deaths. | Total Age. | Average. |
|---|---|---|---|
| 22-32 | 81 | 2,242 | 27.67+ |
| 33-43 | 63 | 2,435 | 38.65+ |
| 44-54 | 87 | 4,262 | 49. |
| 55-65 | 110 | 6,555 | 59.13+ |
| 66-76 | 114 | 8,093 | 70.99+ |
| 77-87 | 52 | 4,182 | 80.42+ |
| 88-100 | 5 | 474 | 94.8 |
| | 512 | )28,243 | |
| | | 55.16 years. | |

As an addenda, let me quote from the statistics as given in 1892 relative to Great Britain,—viz., that the average life in Great Britain is nine years longer than fifty years ago.

Furthermore, I quote from a speech of Dr. J. Y. Crawford, delivered at the World's Columbian Dental Congress, in which he claimed that "modern dentistry has exerted greater potency than all others in bringing about this felicitous [longevity] result."

"No intelligent observer will deny that the great awakening of civilized men, particularly noticeable in the United States, as to the importance of preserving the natural teeth has, in the last half-century, been a perceptible influence in bringing about the increased longevity alluded to, and that it will be still greater in the future."

This will also apply to dentists, for naturally their teeth are cared for as well as those of the laity.

In closing let me digress somewhat and call attention to the rapid discoveries in scientific research, which may well cause us to pause and give thought to almost the last act at the close of the nineteenth century, wherein for the first time we are confronted—through certain chemical experiments—by the "scientific discovery of the principle of life," which makes possible for man to create life by artificial means. And in this connection why not go further and believe with the Christian Scientist that the human species may be propagated, not necessarily by natural process, but by the simple one of *thinking?* And why may we not then discard the Brown-Séquard "elixir of life," and trust to the lengthening of our allotted span by chemical or other processes to be discovered? Surely, then, we will have reached the millenium.

